# Genetic Associations with Aging Muscle: A Systematic Review

**DOI:** 10.3390/cells9010012

**Published:** 2019-12-19

**Authors:** Jedd Pratt, Colin Boreham, Sean Ennis, Anthony W. Ryan, Giuseppe De Vito

**Affiliations:** 1 Institute for Sport and Health, University College Dublin, Dublin, Ireland; colin.boreham@ucd.ie (C.B.); giuseppe.devito@ucd.ie (G.D.V.); 2 Genomics Medicine Ireland, Dublin, Ireland; sean.ennis@ucd.ie (S.E.); anthony.ryan@genomicsmed.ie (A.W.R.); 3 UCD ACoRD, Academic Centre on Rare Diseases, University College Dublin, Dublin, Ireland; 4 Department of Biomedical Sciences, University of Padova, Via F. Marzolo 3, 35131 Padova, Italy

**Keywords:** genotype, genetic variation, muscle phenotypes, sarcopenia, aging

## Abstract

The age-related decline in skeletal muscle mass, strength and function known as ‘sarcopenia’ is associated with multiple adverse health outcomes, including cardiovascular disease, stroke, functional disability and mortality. While skeletal muscle properties are known to be highly heritable, evidence regarding the specific genes underpinning this heritability is currently inconclusive. This review aimed to identify genetic variants known to be associated with muscle phenotypes relevant to sarcopenia. PubMed, Embase and Web of Science were systematically searched (from January 2004 to March 2019) using pre-defined search terms such as “aging”, “sarcopenia”, “skeletal muscle”, “muscle strength” and “genetic association”. Candidate gene association studies and genome wide association studies that examined the genetic association with muscle phenotypes in non-institutionalised adults aged ≥50 years were included. Fifty-four studies were included in the final analysis. Twenty-six genes and 88 DNA polymorphisms were analysed across the 54 studies. The *ACTN3*, *ACE* and *VDR* genes were the most frequently studied, although the *IGF1/IGFBP3*, *TNFα*, *APOE*, *CNTF/R* and *UCP2/3* genes were also shown to be significantly associated with muscle phenotypes in two or more studies. Ten DNA polymorphisms (rs154410, rs2228570, rs1800169, rs3093059, rs1800629, rs1815739, rs1799752, rs7412, rs429358 and 192 bp allele) were significantly associated with muscle phenotypes in two or more studies. Through the identification of key gene variants, this review furthers the elucidation of genetic associations with muscle phenotypes associated with sarcopenia.

## 1. Introduction

Sarcopenia refers to the progressive deterioration in skeletal muscle mass, strength and physical function with advancing age [1]. The simultaneous presence of low muscle strength, muscle mass and/or physical function forms the diagnostic basis of the recommendations from the European Working Group on Sarcopenia in Older People [2]. These criteria are strong predictors of a multitude of adverse health outcomes, such as cardiovascular disease [3], functional disability [4], fall incidence [5], hospitalisation [6], stroke [7] and mortality [8]. Up to 10% of individuals aged 60–69 years are affected by sarcopenia, with this proportion rising considerably to 40% for adults over 80 years of age [9,10]. The fundamental loss of independence and susceptibility to additional diseases caused by sarcopenia also places a significant burden on public health systems worldwide. This burden is anticipated to grow considerably in coming decades, in line with increases in longevity and the consequent rise in the proportion of elderly [11]. Thus, the consequences of age-related muscle deterioration will become increasingly relevant globally.

While sarcopenia is generally more prevalent among individuals over the age of 60, strong evidence suggests that pronounced changes in muscle tissue begin from around 50 years of age [12]. From this age, muscle mass and strength begin to deteriorate at an annual rate of 1–2% and 1.5–5% respectively [12,13,14]. Developing an understanding of why and how skeletal muscle deteriorates from this age will be critical to reducing the burden of sarcopenia for patients as well as public health systems.

Currently, it is known that inter-individual variation in muscle phenotypes may be attributed to genetic factors, environmental factors and/or, gene-environment interactions [15,16]. While environmental factors such as physical activity, protein intake [17], sleep quality [18], smoking status [15] and alcohol consumption [19] have been shown to affect muscle phenotypes, heritability studies have highlighted the importance of genetic factors in determining inter-individual variability in skeletal muscle traits [20,21]. These studies have found that genetic factors account for 46–76% and 32–67% of fat-free mass (FFM) and muscle strength variability, respectively [20,21]. Additional longitudinal studies have observed heritability estimates of 64% for change in muscle strength with advancing age [22]. However, while the overall heritability of skeletal muscle phenotypes is well established, the genetic mechanisms underpinning this heritability remain unclear.

Thus, developing a deeper understanding of genetic associations underpinning skeletal muscle phenotypes is of paramount importance in the development of effective treatment interventions to manage age-related changes in muscle structure and function. Furthermore, understanding the genetic mechanisms regulating muscle accrual and loss will help facilitate early screening for susceptibility to sarcopenia, which could allow for preventative measures to be implemented prior to predicted muscle degradation.

Therefore, the purpose of this systematic review was to identify and synthesize the genetic variants associated with muscle phenotypes relevant to sarcopenia in humans.

## 2. Materials and Methods

Reporting followed the Preferred Reporting Items for Systematic Reviews and Meta-Analyses (PRISMA) statement [23].

### 2.1. Literature Search and Eligibility Criteria

#### 2.1.1. Inclusion and Exclusion Criteria

To be included in this review, studies had to meet the following criteria:

1. Published between January 2004 and March 2019.
2. Full English text available.
3. Participants must be non-institutionalised human adults, aged 50 years or above.
4. Subjects must have been free from any significant cardiovascular, metabolic or musculoskeletal disorders at the time of the study.
5. Candidate gene association study or genome wide association study (GWAS).

#### 2.1.2. Search Strategy

A systematic literature search of three online databases, PubMed, EMBASE and Web of Science, was conducted on 18 March 2019, for the period between January 2004 and March 2019. This time limit ensured the inclusion of the most pertinent literature. Search terms were selected based off the PEO framework and combined using Boolean operators (“AND”, “OR”). Filters were used to limit results to those using human subjects, written in the English language and published within the desired time-frame. The search strategy used was as follows: (“ageing” OR “aged” OR “elderly” OR “older persons” OR “community dwelling”) AND (“sarcopenia” OR “skeletal muscle” OR “muscle phenotype” OR “muscle mass” OR “muscle atrophy” OR “muscle strength” OR “grip strength” OR “physical performance” OR “muscle quality” OR “lean mass”) AND (“single nucleotide polymorphism” OR “genetic polymorphism” OR “allele” OR “genetic variation” OR “gene variant” OR “mutation” OR “genes” OR “chromosome” OR “genetic predisposition” OR “genetic susceptibility”) AND (“genetic association studies” OR “genome-wide association study” OR “GWAS” OR “candidate gene study” OR “genotype” OR “haplotype” OR “heritability”). The scope of the online search was further expanded by assessing bibliographic references of the eligible full text articles for relevant studies.

### 2.2. Study Selection and Data Extraction

Following the removal of duplicates, titles and abstracts were screened for relevance to the scope of this review. To determine inclusion in this review, the full text of every potentially relevant article was scrutinised for overall content and compliance with the eligibility criteria outlined above. The following data were extracted from each eligible article: authors, year of publication, study design, studied population (number, ethnicity, nationality, sex), gene name, polymorphism, muscle phenotype, main findings of the study.

### 2.3. Phenotypes

Phenotypic outcomes included in this systematic review were skeletal muscle mass, muscle strength, physical function and sarcopenia prevalence.

### 2.4. Quality Assessment

The quality and risk of bias of the included studies were assessed using the Quality of Genetic Association Studies (Q-Genie) tool [24]. The Q-Genie tool consists of 11 items that cover the following areas: “rationale for study”, “selection and definition of outcome of interest”, “selection and comparability of comparison groups”, “technical classification of the exposure”, “non-technical classification of the exposure”, “other source of bias”, “sample size and power”, “a priori planning of analysis”, “statistical methods and control for confounding”, “testing of assumptions and inferences for genetic analysis” and “appropriateness of inferences drawn from results”. Each area was rated using a 7-point Likert scale (“1 = poor”; “2”, “3 = good”; “4”, “5 = very good”; “6”, “7 = excellent”). The overall quality of the included articles was classified by collating the scores for each theme. Studies with control groups were classified as “poor quality” if the score was ≤ 35, “moderate quality” if the score was > 35 and ≤ 45, and “good quality” if the score was > 45. For studies without control groups, scoring ≤ 32, > 32 and ≤ 40, and > 40 reflected classifications of “poor quality”, “moderate quality” and “good quality”, respectively.

## 3. Results

### 3.1. Search Strategy

**Figure 1:**
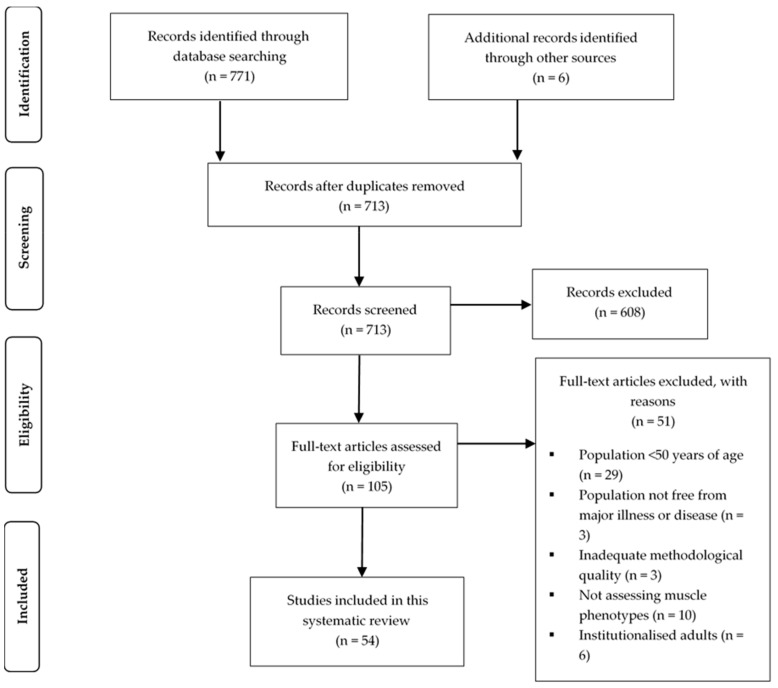
PRISMA flow chart presenting the identification and selection process of articles.

The systematic search of the online databases identified 771 papers. Following the addition of filters, removal of duplicates and screening for eligibility, 48 studies remained. Six additional articles were retrieved through the manual search of reference lists, leaving a total of 54 articles to be included in this systematic review. Figure 1 highlights the identification and selection process in accordance with the PRISMA statement.

### 3.2. Quality Assessment

A detailed quality classification for each article is displayed in Table 1. Studies scored between 33 and 50 in the Q-Genie checklist. For studies with control groups (*n* = 12), five were classified as “moderate quality” and seven as “good quality”. For non-control group studies (*n* = 42), 17 were classified as “moderate quality” and 25 as “good quality”.

### 3.3. Study and Subject Characteristics

Of the 54 studies included in this systematic review, 35 were cross sectional studies while the remaining 19 were longitudinal. A comprehensive description of the characteristics of the cross-sectional studies are presented in Table 2. Of the longitudinal studies, 11 were intervention studies while 8 were observational follow-up studies. The average intervention length was 21.3 weeks (range 10–72 weeks) while the average follow-up was 4.2 years (range 1–10 years). Table 3 presents a detailed description of the characteristics of the longitudinal studies. Out of the 54 studies, 53 were candidate gene association studies and the remaining article was a genome-wide association study.

A total of 38,112 subjects participated across the 54 studies. Of these, 24,890 (65.3%) were female and 13,222 (34.7%) were male. Thirty-two studies included Caucasians, 13 assessed Asian subjects and the remaining nine studies included Hispanic and African-American participants. As described in the inclusion criteria, all subjects were older than 50 years of age. Thirteen studies included subjects over 50 years of age, 22 studies recruited subjects over 60 years of age and 19 studies included individuals aged 70 years or older.

### 3.4. Phenotypes and Genotypes

Of the included studies, 26 reported skeletal muscle mass outcomes, 39 studies included muscle strength testing, 27 articles analysed physical function and six examined sarcopenia prevalence. A full description of the phenotypic outcomes in each study are presented in Table 2 and Table 3.

In total, 88 DNA polymorphisms in or near to 26 different genes were analysed across the 54 studies included in this review. The Alpha-actinin 3 (*ACTN3*), Angiotensin Converting Enzyme (*ACE*), and Vitamin D Receptor (*VDR*) genes were the most frequently researched, present in 14, 13 and nine articles, respectively. For clarity and ease of interpretation in the present review, genes are categorised into three main groups: hormone genes, growth factor and cytokine genes and structural and metabolic genes.

### 3.5. Synthesis of Results

#### 3.5.1. Hormone Genes

##### *VDR* 

Nine studies analysed the association between *VDR* polymorphisms and muscle phenotypes. The first, conducted in 2004 by Roth et al. [67], highlighted a significant association between the rs2228570 (*Fok1*) polymorphism and FFM. Male FF homozygotes had significantly less FFM, appendicular fat-free mass (AFFM) and skeletal muscle index (SMI) compared to f allele carriers (*p* = 0.002, *p* = 0.009, *p* = 0.001 respectively). Furthermore, when classified as sarcopenic, FF carriers were at a two-fold higher risk of being sarcopenic when compared to carriers of the f allele (*p* = 0.03). Hopkinson et al. [45] also found significant interactions between the rs2228570 polymorphism and muscle phenotypes with male FF homozygotes displaying significantly lower knee extensor (KE) strength than f allele carriers (*p* = 0.007). Similarly, Xia et al. [74] found subjects carrying one or more F alleles to have significantly lower handgrip (HG) strength, and FFM (*p* = 0.03, *p* = 0.04 respectively). Furthermore, these individuals had a significantly higher risk of sarcopenia than ff homozygotes (*p* = 0.03). In contrast, a study conducted by Gussago et al. [42] found FF homozygotes to have significantly greater HG strength than f allele carriers (*p* = 0.021).

Significant associations were also identified between the rs1544410 (*Bsm1*) polymorphism and muscle performance phenotypes although, in keeping with the above findings, results were conflicting. In a study conducted by Onder et al. [62], bb homozygotes were significantly less likely to fall than carriers of the B allele (*p* = 0.02). Similarly, in 2010, Barr et al. [27] found females who were homozygous for the b allele to have a significantly lower risk of falling than Bb/BB carriers. These individuals also performed significantly better in the rise from chair and power tests when compared to carriers of B allele (*p* = 0.03, *p* = 0.044 respectively). Contrarily to the above studies, Bahat et al. [26] found KE strength to be significantly higher in BB homozygotes compared to carriers of one or more b alleles (*p* = 0.038).

Additional *VDR* polymorphisms rs7136534 and rs7975232 (*Apa1*) were significantly associated with fall incidence and HG strength respectively (*p* = 0.002, *p* < 0.05) [28,73]. No significant associations were found for the rs731236 (*Taq1*) polymorphism.

##### Other Genes

Genes encoding the androgen receptor (*AR*), thyrotropin-releasing hormone receptor (*TRHR*) and receptor activity-modifying protein 3 (*RAMP3*) were also shown to associate significantly with skeletal muscle traits (Table 2 and Table 3) [57,66,72].

#### 3.5.2. Growth Factor and Cytokine Genes

##### *IGF1* and *IGFBP3* 

The interaction between the Insulin-like Growth Factor 1 (*IGF1*) gene and muscle phenotypes was particularly evident in the intervention studies (Table 3). Both Kostek et al. [50] and Hand et al. [43] demonstrated that carriers of one or more 192 alleles achieved significantly greater KE strength improvements in response to resistance training (RT), compared to non-carriers (*p* = 0.02, *p* < 0.01). However, in a cross-sectional study conducted by Mora et al. [61], no significant differences were observed in muscle strength between carriers and non-carriers of the 192 allele (*p* = 0.024).

Significant associations were also noted for polymorphisms rs35767 of the *IGF1* gene and rs2854744 of the Insulin-like Growth Factor Binding Protein 3 (*IGFBP3*) gene. Kostek et al. [51] observed black females carrying the CC genotype of the rs35767 polymorphism to have significantly less total FFM and muscle cross sectional area (CSA) than TT carriers (both *p* < 0.05). Furthermore, male CC homozygotes performed significantly worse in the single leg chair stand test than carriers of the T allele (*p* < 0.05). In a study conducted by Yang et al. [75], CC carriers of the rs2854744 polymorphism had a 4.3 times higher risk of having low SMI compared to AA carriers (*p* < 0.05).

##### *CNTF* and *CNTFR* 

Two studies examined the Ciliary Neurotrophic Factor (*CNTF*) and Ciliary Neurotrophic Factor Receptor (*CNTFR*) genes (Table 2). In 2006, Arking et al. [25] observed five DNA polymorphisms (rs948562, rs1800169, rs550942, rs4319530, rs1938596) of the *CNTF* gene to be significantly associated with HG strength (*p* < 0.05). Further haplotype analysis revealed the null allele (A) of rs1800169 to fully explain this relationship under a recessive model. Individuals homozygous for the A allele had 3.8 kg lower HG strength than G allele carriers (*p* < 0.01). Interestingly, De Mars et al. [36] found only G/A carriers of the rs1800169 polymorphism to have significantly lower KE strength than G/G or A/A carriers (*p* = 0.0229). Additionally, male T allele carriers of the rs3808871 polymorphism produced significantly higher KE and knee flexor (KF) isometric torque at 120° when compared to CC homozygotes (*p* < 0.05). Furthermore, females who carried the T allele of the rs2070802 polymorphism produced greater KF concentric torques than the A/A homozygotes (*p* = 0.04).

##### *TNFα* 

Three studies were included in this review which investigated the Tumour Necrosis Factor Alpha (*TNFα*) gene, each with significant findings. In 2013, Pereira et al. [65] observed that G allele homozygotes of the rs1800629 polymorphism achieved significantly faster timed up and go (TUG) test results in response to 10 weeks of RT compared to A allele carriers (*p* < 0.001). Additionally, Tiainen et al. [69] found the A allele of the rs361525 polymorphism to be associated with a significantly better physical performance level compared to GG homozygotes (*p* < 0.001). Finally, Li et al. [53] highlighted the interaction between the A allele of the rs1799964 polymorphism with either the G allele of the Tumour Necrosis Factor Beta (*TNF-β*) rs909253 polymorphism or the A allele of the *TNF-β* rs1041981 polymorphism to result in significantly lower handgrip strength among females (*p* = 0.005, *p* = 0.006 respectively).

##### Other Genes

Polymorphisms rs2276541 of the activin A type IIB receptor (*ACVR2B*) gene, rs3807987 of Caveolin 1 (*CAV1*) gene and rs1805086 of the Myostatin (*MSTN*) gene were all significantly associated with FFM (Table 2) [41,49,56].

#### 3.5.3. Structural and Metabolic Genes

##### *ACTN3 (The Sprint Gene)* 

In this review, fourteen studies were included which examined the association between the *ACTN3* rs1815739 (*R577X*) polymorphism and skeletal muscle phenotypes. Carrying the X allele was often associated with lower baseline muscle strength and function (Table 2). For example, in a study conducted by Kikuchi et al. [48], homozygosity for the X allele was associated with significantly poorer performance in the chair stand test compared to RR carriers (*p* = 0.024). Ma et al. [58] also found XX homozygotes to perform significantly worse in HG strength (*p* = 0.012), 5 m walk (*p* = 0.011) and TUG (*p* = 0.039) tests and to also have a significantly higher frailty index (*p* = 0.004). Similar results were observed by Judson et al. [46] in a group of 4163 females where RX and XX genotypes were significantly associated with fall incidence (*p* = 0.049, *p* = 0.02 respectively). In contrast, Delmonico et al. [37] found female XX homozygotes to have significantly higher absolute and relative KE peak power and peak velocity than carriers of the R allele (*p* < 0.05).

Individuals carrying the XX genotype were also shown to have significantly lower improvements in one repetition maximum (1RM) bench press and leg extension, vertical jump and sit-to-stand performance in response to speed and power training when compared to RR carriers (all *p* < 0.05) [63]. Pereira et al. [64] also demonstrated XX carriers to have significantly poorer improvements in 10 m sprint times in response to high speed and power training compared to RR homozygotes (*p* = 0.044). Similarly, female XX carriers were observed to have significantly lower improvements in relative KE peak power following RT compared to RR homozygotes (*p* = 0.02) [37]. In the male population, change in absolute KE peak power post RT approached significance when comparing RR and XX genotypes (*p* = 0.07) [37]. In contrast to the above studies, Delmonico et al. [38] found male XX homozygotes had a significantly greater increase in 400 m walk time when compared to RX/RR carriers (*p* = 0.03).

In a study conducted by Zempo et al. [77] XX homozygotes were observed to have significantly lower thigh muscle CSA compared to RR carriers (*p* = 0.04). Interestingly, in a secondary analysis comparing a middle age group with an old age group, XX homozygosity was only associated with low thigh muscle CSA in the old age group (*p* < 0.05), suggesting that the influence of ACTN3 deficiency is heightened with age [78]. Similar results were noted in 2017 by Cho et al. [32], where sarcopenia prevalence was significantly associated with the XX genotype (*p* = 0.038). In contrast, Lima et al. [54] found X allele carriers to have significantly more relative total FFM than RR homozygotes (*p* = 0.04).

Three studies found no significant differences in muscle phenotypes between *ACTN3* rs1815739 genotypes [30,39,59].

##### *ACE* 

The relationship between the *ACE* rs1799752 (insertion/deletion) polymorphism and skeletal muscle traits has been extensively investigated since the original study of Montgomery et al. in 1998 [79]. Thirteen articles are included in this review. Firstly, Charbonneau et al. [31] found that carriers of the DD genotype had significantly greater total FFM (*p* < 0.05) and lower limb muscle volume (*p* = 0.01) than II homozygotes. Similarly, in a study of 246 Brazilian females, Lima et al. [54] noted DD homozygotes to have a significantly greater SMI than I allele carriers (*p* = 0.044). These findings were further strengthened by Da Silva et al. [34], who demonstrated sarcopenia prevalence to be significantly higher in II genotype carriers compared to D allele carriers (*p* = 0.015) (Table 2). Interestingly, Lima et al. [54] showed that in response to RT, only *ACE* II homozygotes significantly increased AFFM (*p* < 0.001).

The II genotype was also associated with lower muscle strength and functional performance. For example, within a group of 431 Japanese individuals, Yoshihara et al. [76] found II homozygosity to be associated with significantly lower HG strength compared to D allele carriers (*p* = 0.004). Homozygosity for the I allele was also shown to associate with significantly poorer performance in the 6-min walking test and 8 ft TUG test (*p* = 0.008, *p* < 0.001 respectively) when compared to ID/DD genotypes. Furthermore, in response to RT, DD carriers achieved significantly greater improvements in 1RM bench press and sit-to-stand performance (*p* = 0.019, *p* = 0.013 respectively) [63]. Giaccaglia et al. [40] also found that DD genotype carriers achieved significantly greater improvements in concentric KE strength in response to RT compared to II homozygotes (*p* < 0.05). Similarly, Pereira et al. [64] observed that DD homozygotes became significantly quicker performing 10 m sprints (*p* = 0.012) compared to II carriers. Buford et al. [29] also reported that a 12-month exercise intervention evoked significant improvements in 400 m walking speed (*p* = 0.018) and short physical performance battery test (SPPB) scores (*p* = 0.015), but only in D allele carriers. Interestingly, II homozygosity was also significantly associated with developing mobility limitation at a 45% faster rate when compared to ID/DD carriers (*p* = 0.01) [52].

As with the *ACTN3* rs1815739 genotypes, three studies found rs1799752 genotypes to have no significant influence on skeletal muscle traits [30,39,59].

##### *APOE* 

Three studies demonstrated significant associations between the Apolipoprotein E (*APOE*) gene and muscle phenotypes (Table 3). A 6-year follow-up study conducted by Melzer et al. [60] found that e4 carriers displayed significantly slower gait speed and chair stand performance (*p* = 0.006, *p* = 0.015 respectively) at baseline and significantly slower chair stand performance (*p* = 0.034) at the end of the 6-year follow-up, compared to e3 carriers. The *APOE* e4 allele was also shown to be associated with a significantly larger decline in HG strength between the ages of 75 and 79 over a 4-year period, compared to non-carriers (*p* = 0.015) [68]. Furthermore, carriers of the e4 allele had significantly lower HG strength at age 79 compared to non-carriers (*p* = 0.006). Interestingly, the effect of the e4 allele on HG strength was significantly larger at age 79 than age 75 (*p* = 0.033), suggesting that the e4 allele becomes increasingly influential with age. In a 3-year follow-up study conducted by Verghese et al. [71], males carrying the e4 allele showed a significantly more rapid decline in gait speed and greater risk of disability than male non-carriers (*p* = 0.04, *p* = 0.007 respectively).

##### *UCP2* and *UCP3*

Three studies reported significant interactions between Uncoupling Proteins 2/3 (*UCP2/3*) polymorphisms and skeletal muscle traits. Firstly, in a group of 432 Caucasians, Crocco et al. [33] found carriers of the CC genotype of the *UCP3* rs1800849 polymorphism to exhibit significantly lower HG strength than carriers of the T allele (*p* = 0.010). Dato et al. [35], then showed that individuals carrying the AA genotype of *UCP3* rs11235972 polymorphism have significantly lower HG strength than GG homozygotes (*p* < 0.001). In 2015, Keogh et al. [47] demonstrated that GG carriers of *UCP2* rs659366 polymorphism perform significantly worse in the 8 ft TUG test compared with AA/GA genotypes (*p* = 0.045). However, post RT intervention, GG homozygotes of *UCP2* rs659366 had the greatest improvements in 8 ft TUG performance (*p* = 0.023).

##### Genome-wide Studies

Other genes that demonstrated significant associations with muscle phenotypes included the PR domain containing 16 (*PRDM16*) gene, Zinc finger protein 295 (*ZNF295*) gene and C2 calcium dependent domain containing 2 (*C2CD2*) gene (Table 2 and Table 3) [44,70].

Moreover, a recent GWAS by Hernandez-Cordero et al. [80] evaluated genetic contribution to ALM in the UK Biobank dataset, comparing middle-aged (38–49 years) and elderly (60–74 years) individuals. A total of 182 genome-wide significant regions, many with multiple variants within them, were associated with ALM in middle-aged individuals. Of these, 78% were also associated with ALM in elderly individuals. Variants at three genes, *VCAM*, *ADAMTSL3* and *FTO*, had previously been associated with lean body mass in the UK Biobank [81]. Hernandez Cortez et al. also confirmed, in vitro, a functional role for *CPNE1* and *STC2* in myogenesis. In addition, the study highlighted five genomic regions, containing multiple genes, that are associated with muscle mass in both mice and humans.

## 4. Discussion

To the best of the authors’ knowledge, this is the first systematic review to collate literature on genetic associations with muscle phenotypes relevant to sarcopenia. To date, most research targeting genetic associations with muscle phenotypes has not focused on elderly subjects, and thus, the genetic mechanisms underpinning the age-related changes in skeletal muscle traits are largely uncharted.

Given that the deterioration of skeletal muscle with advancing age can have profound consequences for patients and public health systems, improving our understanding of how genes influence this process is of paramount importance. This review has enhanced our knowledge surrounding the key genes and gene variants that may prove crucial in further developing our understanding of the pathogenesis of sarcopenia and improving prognosis and treatment interventions alike.

### 4.1. Summary of Findings

The systematic literature search identified 24 genes and 46 DNA polymorphisms whose expression was significantly associated with muscle phenotypes in older adults. Ten of these DNA polymorphisms (rs154410, rs2228570, rs1800169, rs3093059, rs1800629, rs1815739, rs1799752, rs7412, rs429358 and 192 bp allele) were significantly associated with muscle phenotypes in two or more studies. The complex and multifactorial mechanisms underpinning muscle regulation suggest that the accrual and loss of muscle mass and muscle strength is not reducible to one single gene or gene variant. The dynamic interactions between inhibitory and promotory pathways within the human body further highlight the importance of a holistic approach when considering genetic associations with skeletal muscle traits.

Nevertheless, the findings of this systematic review demonstrate that the most compelling current evidence in the field exists for the *ACTN3*, *ACE* and *VDR* genotypes.

#### 4.1.1. ACTN3 (The Sprint Gene)

The *ACTN3* gene is among the most extensively researched genes in relation to muscle phenotypes, and appeared most frequently within this review. The ACTN3 protein encoded by the *ACTN3* gene forms an integral part of the sarcomere Z-line in fast twitch muscle fibres and further aids in coordinating myofiber contractions [82,83]. Up to 20% of humans are deficient in this protein, due to homozygosity for the premature stop codon at the rs1815739 polymorphism [84]. This significant proportion of ACTN3 deficiency among the population suggests that X allele status is a key factor in variability in muscle phenotypes. In this regard, much of the research surrounding the *ACTN3* genotype has focused on athletic performance [85]. Association studies have repeatedly found reduced X allele frequency among elite sprint/power athletes [85,86,87]. This suggests that the presence of ACTN3 is crucial for the optimal generation of force. Considering that fast twitch muscle fibres are particularly susceptible to age-related atrophy [88], it is plausible that regulation of this protein may also be an important factor in understanding age-related changes in muscle phenotypes. To date, however, limited research has been conducted within elderly populations, with the result that the true impact of the *ACTN3* gene on age-related changes in muscle phenotypes remains inconclusive. Despite this, fourteen of the studies included in this review examining the *ACTN3* genotype reported promising findings. Carriers of the X allele were often found to display lower skeletal muscle mass, strength and functional abilities. This was particularly evident among the Asian population. All five cross-sectional studies that examined Asian participants found significant associations between X allele status and muscle phenotypes [32,48,58,77]. No such association was found in the other three cross-sectional studies that targeted Caucasian individuals [30,39,59], therefore suggesting ethnicity may determine the degree to which *ACTN3* genotypes effect aging muscle. This coincides with existing research whereby X allele frequency and fast twitch fibre composition have been shown to vary across different ethnic groups [89,90,91,92]. The Asian population have the highest frequency of the X allele [89], while having the lowest percentage of fast twitch muscle fibres [90,91,92], two likely contributing factors in the ethnic group having the highest sarcopenia prevalence globally [93]. Unlike above, X allele status was significantly associated with training adaptation within Caucasian, North-American and South-American individuals. Thus, the inconsistencies within this review highlight the need for future research to provide clarification on how ethnicity, *ACTN3* genotypes and muscle phenotypes are associated within the elderly.

#### 4.1.2. ACE

Like the *ACTN3* gene, the *ACE* gene has been widely researched within athletic populations, and knowledge within older populations is limited. There are, however, compelling molecular pathways controlled by the *ACE* gene that suggest its importance in age-related changes in muscle phenotypes. The ACE is expressed by skeletal muscle endothelial cells, and catalyses the production of angiotensin II, known to enhance skeletal muscle hypertrophy [94,95]. To date, research in relation to muscle phenotypes has centred around the *ACE* rs1799752 polymorphism. The D and I alleles have been associated with higher and lower ACE activity respectively [96,97,98]. The D allele is suggested, therefore, to associate with greater muscle performance. To support this hypothesis, recent studies have focused on the rs1799752 polymorphism in elite athletes, with interesting findings. The I allele has been repeatedly associated with endurance performance, while the D allele associates with strength/power capabilities [99,100]. Findings from this systematic review further strengthen these observations. The D allele was consistently associated with higher baseline muscle strength and functional performance, as well as greater improvements in muscle strength and function in response to RT. Evidence of the association between the *ACE* rs1799752 polymorphism and muscle mass is less definitive. While the D allele was often associated with greater amounts of FFM, contradictory findings were also in evidence, and thus, further research is needed in this area to reach a consensus. Like with *ACTN3* genotypes, frequency of the I and D allele of the *ACE* gene are highly determined by ethnic background. Asians have been shown to have the highest frequency for the undesirable I allele [101], while African-American have the lowest [101], aligning with global sarcopenia prevalence estimates where Asians and African-Americans have the highest and lowest risk respectively [93]. While evidence in this review is insufficient in highlighting a true ethnic impact on the association between *ACE* genotypes and aging muscle phenotypes, the disparity in allele frequency among different ethnicities is promising.

#### 4.1.3. VDR

The true significance of the association between the *VDR* gene and muscle phenotypes is currently unknown. While the *VDR* gene has been extensively researched, findings are often contradictory. Furthermore, due to its crucial role in regulating calcium absorption, much of the existing research has focused on the association between *VDR* genotypes and bone health [102]. However, the *VDR* gene is also known to stimulate changes in muscle protein synthesis through its key regulatory role in the transcription of messenger RNA [103], and thus, the potential of the *VDR* gene as a candidate gene for muscle phenotype associations has been suggested. More specifically, the rs2228570 polymorphism is the only known VDR polymorphism where variation results in structural changes within the VDR protein due to differences in translational initiation sites [104]. The *VDR* f allele results in a full length VDR protein of 427 amino acids [105], while a *VDR* F allele results in a truncated VDR protein with three amino acids less [106]. Interestingly, three of four studies that examined the rs2228570 polymorphism in this review found F allele carriers to perform significantly worse across a range of muscle phenotypes [45,67,74], suggesting the potential importance of the rs2228570 polymorphism.

While compelling evidence exists supporting the importance of the *VDR* gene for muscle phenotypes, many studies have failed to replicate earlier results, and thus, the strength of this association remains to be established [107,108]. Unlike for *ACTN3* and *ACE* polymorphisms, evidence of an ethnic influence on *VDR* polymorphism frequency is conflicting [109,110]. As with most genetic association studies, much of the research surrounding *VDR* polymorphisms and muscle phenotypes has been conducted using Caucasian subjects. Only nine articles examining *VDR* genotypes were included in this review, seven of which focused on Caucasian individuals [26,27,28,42,45,62,67]. Furthermore, as with the *ACTN3* and *ACE* genes, limited research has been conducted within an elderly population, further limiting the transferability of findings for older adults.

#### 4.1.4. Other Genes of Interest

Other genes with convincing molecular pathways and findings, that warrant future investigation include the *IGF1/IGFBP3*, *TNFα*, *APOE*, *CNTF/R* and *UCP2/3* genes.

#### 4.1.5. IGF1 and IGFBP3

The *IGF* family of genes encode peptides that are crucial in regulating cell proliferation, apoptosis and differentiation [111]. The mitogenic effect of IGF1 is integral to the facilitation of growth in multiple tissues, including skeletal muscle [112]. Considering that advancing age is associated with a decline in circulating IGF1 levels, the *IGF1* gene is a likely candidate to effect muscle phenotypes among the elderly [113]. The current review found significant associations between *IGF1* variants and skeletal muscle mass and strength. Associations were particularly convincing in longitudinal studies, suggesting that the *IGF1* 192 polymorphism may be particularly influential in the strength-training response of skeletal muscle phenotypes as opposed to baseline measurements.

The function of IGF1 is mediated through interactions with binding proteins, mainly, IGFBP3. Research has demonstrated that IGFBP3 is the most prolific potentiator of IGF1, therefore suggesting its importance in explaining inter-individual variation in muscle phenotypes [114]. While only Yang et al. [75] have investigated the impact of the *IGFBP3* gene in an elderly population, the significant findings of that study combined with the relevant gene mechanisms warrants future research.

#### 4.1.6. TNFα

Like the *IGF* family, the *TNFα* gene aids in the regulation of a multitude of biological processes such as cell proliferation, differentiation and apoptosis, and is thus an important candidate gene for aging skeletal muscle [115]. TNFα is also known to be an integral mediator of the inflammatory response to muscle damage [116]. Considering that inflammation is a vital response to RT in facilitating muscle regeneration, the *TNFα* gene is likely to affect the response of skeletal muscle tissue to RT [117]. This is supported by the findings of Pereira et al. [65] who observed that *TNFα* genotypes associate significantly with TUG performance adaptation. While Tiainen et al. [69] also highlighted significant cross-sectional associations, these were based on self-reported measures and should be interpreted with caution. Thus, longitudinal studies focusing on RT response of skeletal muscle may prove most beneficial in understanding the effect of *TNFα* genotypes on the aging muscle.

#### 4.1.7. APOE

APOE protein encoded by the *APOE* gene, is involved in lipid metabolism and is a well-established risk factor for Alzheimer’s disease and various other aging disorders such as cardiovascular disease, atherosclerosis, stroke and impaired cognitive function [118]. Considering the associations between muscle phenotypes such as HG strength and these disorders, research has begun to investigate the relationship between the *APOE* gene and skeletal muscle traits. The gene has three common alleles (e2, e3 and e4), with e2 and e4 carriers having the lowest and highest risk of developing such aging disorders respectively [119]. As a result, much of the research in relation to skeletal muscle has centred around the e4 allele. The e4 allele was consistently associated with unfavourable skeletal muscle traits within this review, and therefore, supports the possibility of *APOE* as a candidate gene for explaining variation in muscle phenotypes with advancing age. Interestingly, like for *ACTN3* and *ACE* genotypes, prevalence of the e4 allele is known to be highly varied among different populations [120]. With only three studies were included in this review, the effect of ethnicity on e4 allele frequency and the resulting association with muscle phenotypes is yet to be confirmed.

#### 4.1.8. CNTF and CNTFR

The *CNTF* and *CNTFR* genes are both mediated through a common signal-transducing component, and thus are often examined in parallel [121]. CNTF, located in glial cells, aids in the promotion of motor neuron survival, and is therefore suggested to limit age-related atrophy of skeletal muscle caused by denervation [122]. The CNTFR is largely expressed in skeletal muscle, promoting research to examine the role of the *CNTF* and *CNTFR* genes in the regulation of muscle phenotypes [123]. To date, however, much of this research has been conducted using rats, with limited research being conducted with human populations. Thus, while the current review has highlighted some significant associations with muscle phenotypes, additional research is required to further understand the mechanisms underpinning this association in humans.

#### 4.1.9. UCP2 and UCP3

Uncoupling proteins (UCPs) are mitochondrial transporters, best known for their involvement in thermogenesis and energy utilisation. As a result, UCPs are most commonly researched in relation to obesity-related phenotypes [124,125]. There is, however, evidence that suggests their importance in regulating muscle phenotypes. UCP2 and UCP3 have both been shown to effect skeletal muscle performance through the inhibition of mitochondrial ATP synthesis [126]. Additionally, *UCP2* and *UCP3* genes serve a key purpose in the protection of cells by attenuating mitochondrial reactive oxygen species (ROS) production, known to exert damaging effects on cells [127]. While loss of skeletal muscle mitochondrial content is known to occur with advancing age [128], evidence suggests UCPs are particularly active in the latter stages of life due to an increase in ROS and the associated rise in mitochondrial superoxide [129]. Therefore, *UCP2* and *UCP3* genes may affect how metabolic function of skeletal muscle is retained during the aging process. While the three studies included in this review found significant associations between *UCP2* and *UCP3* variants and muscle phenotypes, other data from human studies are scarce and as a result, the strength of this association remains to be elucidated.

### 4.2. Strengths and Limitations

This is the first systematic literature review to explore the genetic association with muscle phenotypes among the elderly. Only healthy subjects were included in the review, allowing for any association to be solely attributed to genotype-phenotype interactions rather than disease. All subjects were over the age of 50 years, ensuring relevance towards developing the understanding of the pathogenesis of sarcopenia. While some methodological weaknesses exist, most studies were well designed and conducted.

Findings within this review were at times conflicting. This incongruity may be partly explained by between-study disparities in methodological aspects such as sample size, subject characteristics and false-positive reporting. Furthermore, not all studies utilised the same measure for each muscle phenotype. For example, muscle strength measured through handgrip or leg extension may lead to different results. Evidently, there is a need for genetic association studies to implement more comprehensive and stringent methodology to maximise the potential of identifying genetic variants relevant to aging muscle phenotypes.

Finally, while not necessarily a limitation of this review itself, the overall lack of research currently available regarding the association between genetic variants and muscle phenotypes within the elderly prevents more definitive inferences to be made. As evidenced in this review, most research to date has focused on European populations, thus limiting the transferability of findings to other ethnic groups. Considering the promising ethnic differences in polymorphism frequency previously highlighted, future genetic studies may benefit from including individuals from a variety of ethnic backgrounds. The distinct lack of GWAS targeting aging muscle phenotypes is also contributive towards the uncertainty surrounding this area. A large body of research has utilised a candidate gene approach. Historically, many candidate gene studies have been statistically underpowered, the replication of findings has been problematic and there has been a suspected bias against publication of negative results, which may lead to conflicting findings [130]. Many of these issues have been overcome by GWAS in large, well characterised cohorts [80,131,132,133]. Therefore, future GWAS may help to further illuminate the genetic basis of aging muscle phenotypes.

## 5. Conclusions

The ability to maintain skeletal muscle mass, strength and function with advancing age is essential in preventing sarcopenia. Thus, the elucidation of the genetic variants associated with these phenotypes is of paramount importance. Evidently, skeletal muscle mass, strength and function are multifaceted characteristics that vary widely among the elderly. While heritability studies have highlighted that significant proportions of this inter-individual variability are determined by genetic factors, the specific genes involved remain mostly unknown.

The genetic association with muscle phenotypes is relatively under-researched, with only a limited number of candidate genes being explored to date. This review identified and systematically compiled the key genes shown to be significantly associated with muscle phenotypes within an elderly population. While relatively few genes have been identified which significantly contribute towards variation in muscle phenotypes, promising findings pointing to more extensive associations exist. Evidence is particularly supportive of the *ACTN3*, *ACE* and *VDR* genes, while the *IGF1/IGFBP3*, *TNFα*, *APOE*, *CNTF/R* and *UCP2/3* genes have also been shown to be significantly associated with skeletal muscle phenotypes in two or more studies.

To conclude, the findings from this review helped to further illuminate the genetic basis of sarcopenia. While the molecular genetic pathways are often compelling, the limited volume of research within this field is as yet insufficient to demonstrate a clear genetic basis for sarcopenia. Future GWAS could facilitate the identification of novel genetic variants that may have key regulatory roles in aging muscle phenotypes. Further still, a more extensive exploration of the candidate genes highlighted in this review should provide further insight into the pathogenesis of sarcopenia and further aid in the development of effective prognosis, preventive and treatment protocols to combat the profound consequences of sarcopenia for patients and health systems worldwide.

## Figures and Tables

**Table 1:** Q-Genie quality assessment scores for the included studies.

Studies	Items											Total
	1	2	3	4	5	6	7	8	9	10	11	
Arkin, et al., 2006. [25]	4	4	N/A	4	4	3	3	5	4	5	4	40
Bahat, et al., 2010. [26]	4	4	3	4	3	3	3	4	5	4	4	41
Barr, et al., 2010. [27]	5	3	N/A	4	5	2	4	5	5	4	5	42
Bjork, et al., 2019. [28]	4	4	N/A	5	4	3	5	5	5	4	4	43
Buford, et al., 2014. [29]	5	4	3	4	5	3	3	4	6	4	6	47
Bustamante-Ara, et al., 2010. [30]	6	5	N/A	6	5	4	2	4	5	3	5	45
Charbonneau, et al., 2008. [31]	5	5	N/A	6	5	4	2	5	5	3	4	44
Cho, et al., 2017. [32]	4	4	N/A	5	4	3	3	3	4	3	4	37
Crocco, et al., 2011. [33]	5	5	N/A	5	4	3	5	5	5	4	3	44
Da Silva, et al., 2018. [34]	5	4	3	5	4	3	2	4	4	3	5	42
Dato, et al., 2012. [35]	6	5	N/A	5	4	3	4	5	4	4	5	45
De Mars, et al., 2007. [36]	6	5	N/A	5	4	3	3	5	5	4	5	45
Delmonico, et al., 2007. [37]	4	4	N/A	2	3	3	2	4	4	3	4	33
Delmonico, et al., 2008. [38]	5	3	N/A	3	4	3	5	4	5	3	4	39
Garatachea, et al., 2012. [39]	6	5	N/A	7	6	4	2	3	5	3	5	46
Giaccaglia, et al., 2008. [40]	5	5	5	5	4	5	3	4	5	5	4	50
Gonzalez-Freire, et al., 2010. [41]	5	5	N/A	5	4	3	2	3	4	4	5	40
Gussago, et al., 2016. [42]	5	6	4	5	4	3	3	5	4	4	5	48
Hand, et al., 2007. [43]	5	4	N/A	6	5	3	3	4	6	3	5	44
Heckerman, et al., 2017. [44]	5	4	N/A	5	5	4	2	4	5	5	4	43
Hopkinson, et al., 2008. [45]	4	5	3	4	4	3	3	3	4	4	4	41
Judson, et al., 2010. [46]	5	4	N/A	5	5	2	4	3	4	4	4	40
Keogh, et al., 2015. [47]	5	4	N/A	5	4	3	2	4	3	4	5	39
Klimentidis, et al., 2016. [48]	4	4	N/A	2	4	4	5	5	5	4	4	41
Kikuchi, et al., 2015. [49]	5	4	3	4	4	4	5	5	5	4	5	48
Kostek, et al., 2005. [50]	5	5	N/A	5	5	3	3	4	5	4	5	44
Kostek, et al., 2010. [51]	5	3	3	5	4	2	3	5	5	4	5	44
Kritchevsky, et al., 2005. [52]	5	3	N/A	5	4	4	5	4	5	4	5	44
Li, et al., 2016. [53]	5	6	N/A	4	4	3	5	4	5	3	4	43
Lima, et al., 2011. [54]	5	3	4	5	3	4	3	4	5	4	5	45
Lin, et al., 2014. [55]	5	4	3	5	3	4	4	5	5	4	5	47
Lin, et al., 2014. [56]	5	5	4	5	4	3	3	5	5	5	4	48
Lunardi, et al., 2013. [57]	4	6	N/A	4	3	3	4	5	4	3	5	41
Ma, et al., 2018. [58]	6	5	N/A	5	5	4	5	4	5	3	4	46
McCauley, et al., 2010. [59]	5	5	N/A	5	3	4	3	4	3	3	5	40
Melzer, et al., 2005. [60]	5	5	N/A	4	2	3	4	4	4	4	5	40
Mora, et al., 2011. [61]	4	3	N/A	4	4	4	3	4	5	4	5	40
Onder, et al., 2008. [62]	5	4	N/A	4	4	3	2	5	3	4	4	38
Pereira, et al., 2013. [63]	5	3	N/A	4	4	4	4	3	4	3	5	39
Pereira, et al., 2013. [64]	6	4	N/A	6	4	4	4	5	5	4	5	47
Pereira, et al., 2013. [65]	6	5	N/A	6	5	3	3	5	5	4	5	47
Prakash, et al., 2019. [66]	4	4	3	5	5	3	4	5	4	5	4	46
Roth, et al., 2004. [67]	5	5	N/A	4	3	3	5	4	5	4	5	43
Skoog, et al., 2016. [68]	6	5	N/A	4	3	3	3	4	4	3	5	40
Tiainen, et al., 2012. [69]	6	5	N/A	4	3	4	2	3	3	3	4	37
Urano, et al., 2014. [70]	5	4	N/A	5	3	4	4	4	5	4	5	43
Verghese, et al., 2013. [71]	5	4	N/A	4	3	3	5	4	4	4	5	41
Walsh, et al., 2005. [72]	5	5	N/A	5	4	4	2	4	5	4	4	42
Wu, et al., 2014. [73]	5	5	N/A	4	4	3	4	4	5	3	4	41
Xia, et al., 2019. [74]	5	5	N/A	4	3	4	4	4	5	3	6	43
Yang, et al., 2015. [75]	5	4	N/A	4	3	4	3	4	5	4	5	41
Yoshihara, et al., 2009. [76]	3	4	N/A	3	3	3	4	3	4	3	3	33
Zempo, et al., 2010. [77]	4	4	N/A	3	3	4	3	4	5	4	4	38
Zempo, et al., 2011. [78]	5	4	N/A	3	3	3	4	4	5	4	4	39

Items: 1: Rationale for study, 2: Selection and definition of outcome of interest, 3: Selection and comparability of comparison groups, 4: Technical classification of the exposure, 5: Non-technical classification of the exposure, 6: Other sources of bias, 7: Sample size and power, 8: A priori planning of analysis, 9: Statistical methods and control for confounding, 10: Testing of assumptions and inferences for genetic analyses, 11: Appropriateness of inferences drawn from results. Scoring: 1 to 7, 1 being poor and 7 being excellent. N/A: not applicable.

**Table 2:** Cross-sectional studies on genetic associations with muscle phenotypes.

Gene	Polymorphism	Population Data	N	Muscle Phenotype	Results	Reference
*Hormone Genes*						
*VDR*	rs2228570 (Fok1) rs1544410 (Bsm1)	Caucasians 46 males and 58 females Mean age 61.8 ± 8.5 years	104	Muscle strength (KE strength)	Individuals homozygous for the F allele of the rs2228570 polymorphism displayed significantly lower KE strength than carriers of ≥ 1 f allele (*p* = 0.007). KE strength did not differ significantly across rs1544410 genotypes.	Hopkinson, et al., 2008. [45]
*VDR*	rs2228570 rs1544410	Caucasians (Italians) 87 males and 172 females Aged ≥ 80 years Mean age 85.0 ± 4.5 years	259	Physical function (fall incidence)	Participants homozygous for the b allele of the rs1544410 polymorphism were significantly less likely to fall than carriers of ≥ 1 B allele (*p* = 0.02). Fall incidence did not differ significantly across rs2228570 genotypes.	Onder, et al., 2008. [62]
*VDR*	rs2228570 rs1544410	Caucasian females (OPUS cohort) Mean age 66.9 ± 7.0 years	2363	Muscle strength (lower limb power) Physical function (fall incidence, rise from chair)	Individuals with a bb genotype of the rs1544410 polymorphism were significantly less likely to fall than carriers of ≥ 1 B allele (*p* = 0.025). These individuals also performed significantly better in rise from chair and lower limb power tests (*p* = 0.03, 0.044 respectively). Fall incidence, muscle power did not differ significantly across rs2228570 genotypes.	Barr, et al., 2010. [27]
*VDR*	rs2228570 rs1544410 rs731236 (Taq1)	Males living in Turkey Aged 65–93 years Mean age 69 ± 6.9 years	120	Muscle strength (KE and KF peak torque)	KE strength was significantly higher in BB homozygotes compared to carriers of ≥ 1 b allele of the rs1544410 polymorphism (*p* = 0.038). No significant associations were found for rs2228570 and rs731236 genotypes.	Bahat, et al., 2010. [26]
*Hormone Genes*						
*VDR*	rs2228570 (Fok1) rs1544410 (Bsm1) rs731236 (Taq1) rs7975232 (Apa1)	Caucasian male centenarians (Italian) Mean age 102.3 ± 0.3 years	120	Muscle strength (HG strength)	FF homozygotes displayed significantly greater HG than individuals with ≥ 1 f allele of the rs2228570 polymorphism (*p* = 0.021). HG did not differ significantly between rs1544410, rs7975232 and rs731236 genotypes.	Gussago, et al., 2016. [42]
*VDR*	rs7136534 rs9729 rs17882106 rs10735810 rs4516035 rs11568820 rs11574024	Males living in Sweden Aged 69–81 years Mean age 75.4 ± 3.2 years	2844	Muscle strength (HG strength) Physical function (fall incidence, 6 m walk test, 20 cm narrow walk test, timed-stand test)	AA homozygotes were significantly less likely to fall compared to carriers of ≥ 1 G allele of rs7136534 (*p* = 0.002). No other significant associations were found between polymorphism and muscle strength or function tasks.	Bjork, et al., 2019. [28]
*VDR*	rs2228570 rs1544410	Caucasian males Aged 58–93 years	302	Body composition (FFM, AFFM, SMI) Muscle Strength (KE torque) Sarcopenia (SMI < 7.26 kg/m^2^)	Men homozygous for the F allele of the rs2228570 polymorphism had significantly less FFM, AFFM and SMI compared to Ff/ff genotypes (*p* = 0.002, 0.009, 0.001 respectively). FF homozygotes also had 2.17-fold higher risk of sarcopenia than carriers of ≥ 1 f allele (*p* = 0.03). No similar associations were found between rs1544410 genotypes.	Roth, et al., 2004. [67]
*Hormone Genes*						
*VDR*	rs7975232 (Apa1) rs1544410 (Bsm1) rs2239185 rs3782905	Taiwanese 215 males and 154 females Mean age 74.4 ± 6.3 years (males) and 71.7 ± 4.7 years (females)	369	Muscle strength (HG strength)	Females carrying the AC genotype of rs7975232 polymorphism had significantly lower HG than CC homozygotes (*p* < 0.05). In both men and women, physical inactivity and the minor allele of each polymorphism were jointly associated with increased risk of low HG.	Wu, et al., 2014. [73]
*VDR*	rs2228570 (Fok1)	Chinese 275 males and 510 females Aged 63.2–72.5 years (males) and 63.1–71.9 years (females)	785	Muscle strength (HG strength) Physical function (4 m gait speed) Body composition (FFM, AFFM, SMI) Sarcopenia (SMI < 7.0 kg/m^2^ for men and < 5.4 kg/m^2^ for women and either low HG < 26 kg for men and < 18 kg for women or low gait speed < 0.8 m/s for both sexes)	Males who were homozygous for the f allele of the rs2228570 polymorphism had significantly greater HG and SMI when compared to carriers of ≥1 F allele (*p* = 0.03, 0.04 respectively). These individuals also had a significantly lower risk of sarcopenia (*p* = 0.03). No similar association was found in the female population.	Xia, et al., 2019. [74]
*AR*	rs3032358 (CAG repeat)	Caucasian males (STORM cohort) Aged 55-93 years Subjects grouped by repeat number (120 males had < 22 and 174 had ≥ 22)	294	Body composition (total FFM and SMI) Muscle strength (KE isometric strength and HG strength)	Men who had ≥22 repeats exhibited significantly greater total FFM and SMI than men with < 22 repeats (*p* < 0.027, < 0.019 respectively). A similar association was not found in females. No significant association was observed between repeat number and muscle strength phenotypes.	Walsh, et al., 2005. [72]
*TRHR*	rs16892496 rs7832552	Brazilian females Aged between 60–82 years Mean age 66.6 ± 5.5 years	241	Body composition (FFM, AFFM and SMI) Muscle strength (KE peak torque) Sarcopenia (SMI < 5.45 kg/m^2^)	Subjects who carried the CC variant of rs16892496 had significantly less AFFM and SMI than AA/AC carriers (*p* < 0.05). No significant differences were observed for rs7832552 variants.	Lunardi, et al., 2013. [57]
*Growth Factor and Cytokine Genes*						
*IGF1*	rs35767	Health ABC study cohort Blacks (533 males and 705 females) Whites (925 males and 836 females) Aged 70–79 years	2999	Body composition (FFM) Muscle volume (quadriceps CSA) Muscle strength (KE and HG strength, elbow flexor MVC and 1RM) Physical function (gait speed and single leg chair stands)	Black females with a CC genotype had significantly less FFM and quadriceps CSA compared to TT counterparts (both *p* < 0.05). White males with a CC genotype performed significantly worse in the single leg chair stands compared to CT counterparts (*p* < 0.05).	Kostek, et al., 2010. [51]
*IGF1*	192 bp allele	Caucasians (Spanish) 144 males and 148 females Mean age 76.7 ± 5.4 years (males) and 77.3 ± 6.4 years (females)	292	Muscle strength (KE isometric strength and HG strength)	No significant associations were observed in either males or females with relation to homozygosity, heterozygosity or non-carrier condition of the 192 bp allele (*p* = 0.24).	Mora, et al., 2011. [61]
*IGF1* *IGFBP3* *IGFBP5*	rs6214 rs35767 rs3110697 rs2854744 rs11977526 rs1978346 rs12474719	Taiwanese 251 males and 221 females Aged ≥ 65 years Mean age 74.7 ± 6.4 years (males) and 72.8 ± 5.5 years (females)	472	Body composition (SMI)	Individuals carrying the CC genotype of rs2854744 had a 4.3-fold risk of having low SMI compared with those with the AA genotype (*p* < 0.05). No other significant associations were observed for the other polymorphisms.	Yang, et al., 2015. [75]
*Growth Factor and Cytokine Genes*						
*CNTF*	rs948562 rs1800169 rs550942 rs4319530 rs1944055 rs2510559 rs2275993 rs1938596	Caucasian females (North American) Aged 70–79 years	363	Muscle strength (KE, HE and HG strength)	5 polymorphisms (rs948562, rs1800169, rs550942, rs4319530, rs1938596) were associated with HG (p <0.05). Haplotype analysis revealed rs1800169 null allele to fully explain relationship with the haplotype and HG under a recessive model, with homozygotes for the null allele exhibiting 3.80kg lower HG (p<0.01).	Arking, et al., 2006. [25]
*CNTF* *CNTFR*	rs1800169 rs3808871 rs2070802 C-174T	Caucasians 99 males and 102 females Aged 60–78 years (males) and 60–80 years (females)	201	Body composition (FFM) Muscle strength (isometric and concentric KE and KF at 60°, 120°, 150°, 180°, 240°)	Females who were G/A heterozygotes for the rs1800169 polymorphism produced significantly lower KE at 150° than both G/G and A/A homozygotes (*p* = 0.0229). Males who carried the T allele of the rs3808871 polymorphism produced significantly higher KE and KF isometric torque at 120° when compared to CC homozygotes (*p* < 0.05). Females who carried the T allele of the rs2070802 polymorphism performed better on KF concentric torques at 60°, 180° and 240° than the A/A homozygotes (*p* = 0.03, 0.04, 0.04 respectively). No significant associations were observed between polymorphisms and FFM.	De Mars, et al. 2007. [36]
*Growth Factor and Cytokine Genes*						
*CRP* *IL6* *TNFα* *ICAM1*	rs1800947 rs2069829 rs361525 rs5498	Danish twins 200 males and 400 females Aged 73–95 years	600	Physical function (self-reported during a 2-hour interview using a 11-item checklist)	Males who carried ≥1 A allele of the TNFα rs361525 polymorphism had a significantly better physical performance level compared to GG homozygotes (*p* < 0.001). No other associations were observed between polymorphisms and physical performance.	Tiainen, et al., 2012. [69]
*CRP* *TNFα* *LTA*	rs2794520 rs1205 rs1130864 rs1800947 rs3093059 rs1799964 rs1800629 rs3093662 rs2239704 rs909253 rs1041981	Taiwanese 251 males and 221 females Aged ≥ 65 years Mean age 74.7 ± 6.4 years (males) and 72.8 ± 5.5 years (females)	472	Muscle strength (HG strength)	In females, the main effect of polymorphisms (rs1800947, rs3093059, rs1799964, rs1800629, rs909253, rs1041981) reflected lower HG. In the male population, polymorphisms (rs1130864, rs2239704) produced the same effect.	Li, et al., 2016. [53]
*CRP*	rs2794520 rs1205 rs1130864 rs1800947 rs3093059	Taiwanese 251 males and 221 females Aged ≥ 65 years Mean age 74.7 ± 6.4 years (males) and 72.8 ± 5.5 years (females)	472	Muscle strength (HG strength)	HG of subjects carrying the CC variant of polymorphisms rs2794520 and rs1205 was lower by 1.24 kg and 1.28 kg, respectively, compared with TT homozygotes. HG was 1.01 kg lower for every additional C allele of rs3093059 polymorphism. Haplotype C-C-C-C-C was significantly associated with lower HG than any other haplotypic formation (*p* = 0.015).	Lin, et al., 2014. [55]
*Growth Factor and Cytokine Genes*						
*CAV1*	rs1997623 rs3807987 rs12672038 rs3757733 rs7804372 rs3807992	Taiwanese 265 males and 237 females Aged ≥ 65 years 327 controls, 56 pre-sarcopenic, 63 sarcopenic, 56 severely sarcopenic	502	Body composition (FFM, AFFM, SMI) Muscle strength (HG strength) Muscle function (15 ft walk test) Sarcopenia (SMI < 6.87 kg/m^2^ and 5.46 kg/m^2^ for males and females, respectively and lowest quintile for muscle strength and function tests)	Subjects carrying ≥ 1 A allele of rs3807987 were at a significantly higher risk of sarcopenia than GG homozygotes (*p* = 0.0235). No other significant associations were observed between the remaining polymorphisms.	Lin, et al., 2014. [56]
*MSTN*	rs1805065 rs35781413 rs1805086 rs368949692 rs143242500	Caucasian nonagenarians 8 males and 33 females Aged 90–97 years	41	Muscle strength (1RM leg press) Physical function (Tinetti scale measured gait and balance, Barthel index) Body Composition (FFM estimated)	Carriers of the rs1805086 KR genotype were associated with lower FFM compared to KK carriers. The RR homozygote was below the 25th sex specific percentile for FFM and functional capacity.	Gonzalez-Freire, et al., 2010. [41]
*ACVR2B*	rs2276541	Hispanic (354) and Non-Hispanic (2406) females Mean age 64.1 ± 7.4 years	2760	Body composition (FFM, AFFM)	Subjects carrying the A allele of rs2276541 had significantly more FFM than G allele carriers (*p* = 0.006).	Klimentidis, et al., 2016. [49]
*Structural and Metabolic Genes*						
*ACTN3* *ACE*	rs1815739 (R577X) rs1799752 (I/D)	Caucasians (Spanish) 8 males and 33 females Aged 90–97 years Mean age 92 ± 2 years	41	Muscle strength (HG strength and 6–7 RM leg press) Physical function (8 m walk test and 4 step stairs test)	Study phenotypes did not differ significantly between ACE or ACTN3 genotypes (all *p* > 0.05).	Bustamante, et al., 2010. [30]
*Structural and Metabolic Genes*						
*ACTN3*	rs1815739 (R577X)	Japanese 183 males and 238 females Aged ≥ 55 years	421	Muscle strength (HG strength) Physical function (chair stand test, 8 ft walking test)	XX homozygotes performed significantly worse in the chair stand test than RR/RX carriers (*p* = 0.024, 0.005 respectively). No significant association was found between ACTN3 genotype and 8 ft walk test or HG.	Kikuchi, et al., 2014. [48]
*ACTN3*	rs1815739	Koreans 62 males and 270 females Aged ≥ 65 years Mean age 74.4 ± 4.6 years (males) and 74.4 ± 6.6 years (females)	332	Body composition (FFM, AFFM, SMI) Sarcopenia (SMI < 7.0 kg/m^2^ and < 5.4 kg/m^2^ for men and women respectively)	Sarcopenia prevalence was significantly associated with RX/XX genotypes (*p* = 0.037, 0.038 respectively). This association remained significant under both a dominant and recessive model (*p* = 0.043, 0.029 respectively).	Cho, et al., 2017. [32]
*ACE*	rs1799752 (I/D)	Brazilians 38 males and 53 females Aged 60–95 years Mean age 70.6 ± 7.2 years	91	Body composition (FFM, AFFM, SMI) Muscle strength (HG strength) Physical function (TUG test) Sarcopenia (based off FFM, muscle strength and physical function)	Sarcopenia prevalence was significantly higher in II genotype carriers compared to individuals with ≥ 1 D allele (*p* = 0.015).	Da Silva, et al., 2018. [34]
*ACTN3* *ACE*	rs1815739 rs1799752	Caucasians (Spanish) 22 males and 59 females Aged 71–93 years Mean age 82.8 ± 4.8 years	81	Muscle strength (HG strength) Physical function (30s chair stand test, Barthel index) Muscle volume (thigh muscle CSA and muscle quality)	No significant associations were noted between any ACE rs1799752 or ACTN3 rs1815739 genotypes and the tested phenotypes in either males or females (*p* > 0.05).	Garatachea, et al., 2012. [39]
*Structural and Metabolic Genes*						
*ACTN3*	rs1815739 (R577X)	Chinese 686 males and 777 females Aged 70–87 years 2 age groups (70–79 years and 80–87 years)	1463	Muscle strength (HG strength) Physical function (TUG, 5m walk test) Frailty measure (frailty index containing 23 variables)	In the 70–79 age group, male XX homozygotes performed significantly worse than RR carriers in HG, 5 m walk test and TUG (*p* = 0.012, 0.011 and 0.039 respectively). Females in this age group who carried the XX genotype had a significantly higher frailty index than RR carriers (*p* = 0.004).	Ma, et al., 2018. [58]
*ACTN3* *ACE*	rs1815739 rs1799752 (I/D)	Caucasian males (British) Aged 60–70 years Mean age 65 ± 3 years	100	Body composition (FFM and thigh FFM) Muscle strength (isometric and isokinetic KE strength) Contractile properties (time to peak tension, half-relaxation time, peak rate of force development)	There were no significant associations between either ACE or ACTN3 genotypes and the studied phenotypes.	McCauley, et al., 2010. [59]
*ACE*	rs1799752	Japanese 228 males and 203 females Aged 76 years	431	Muscle strength (HG strength, isokinetic KE) Physical function (10 s maximal stepping rate, single leg standing time with eyes open, maximum walking speed over 10 m)	Individuals homozygous for the I allele had significantly lower HG than carriers of the D allele (*p* = 0.004). Although not significant, the ACE rs1799752 polymorphism was also positively associated with 10 m maximum walking speed.	Yoshihara, et al., 2009. [76]
*ACTN3*	rs1815739	Japanese females Aged 50–78 years Mean age 64.1 ± 6 years	109	Body composition (mid-thigh CSA) Physical function (physical activity was measured using an uniaxial accelerometer)	Thigh muscle CSA was significantly lower in XX homozygotes compared to RX/RR carriers (*p* = 0.04). Physical activity did not significantly differ between genotypes.	Zempo, et al., 2010. [77]
*Structural and Metabolic Genes*						
*ACTN3*	rs1815739 (R577X)	Japanese females Middle aged group (*n* = 82) mean age 50.6 ± 0.9 years Older group (*n* = 80) mean age 66.8 ± 0.5 years	162	Body composition (mid-thigh CSA) Physical activity (physical activity was measured using an uniaxial accelerometer)	In the middle-aged group, no association was observed between ACTN3 genotypes and thigh muscle CSA or physical activity. In the older group, XX homozygotes had significantly lower thigh muscle CSA than RX/RR carriers (*p* < 0.05).	Zempo, et al., 2010. [78]
*UCP3*	rs1800849 rs15763	Caucasians (Italians) 221 males and 211 females Aged 65–105 years Mean age 73.37 ± 7.46 years (males) and 73.37 ± 7.69 years (females)	432	Muscle strength (HG strength)	Carriers of the CC genotype of rs1800849 exhibited significantly lower HG than CT/TT genotypes (*p* = 0.010). No significant association was observed between rs15763 genotypes and HG.	Crocco, et al., 2011. [33]
*UCP3*	rs11235972 rs1685354 rs3781907 rs647126	Caucasians (Danish 1905 cohort) 265 males and 643 females Aged 93 years	908	Muscle strength (HG strength)	Individuals carrying the AA genotype of rs11235972 showed significantly lower HG than GG homozygotes (*p* < 0.001). Subjects carrying a GA genotype of rs1685354 displayed significantly greater HG than AA homozygotes (*p* = 0.016).	Dato, et al., 2012. [35]
*PRDM16*	rs12409277	Japanese females Mean age 65.1 ± 9.4 years	1081	Body composition (total FFM%)	Individuals who carried CT/CC variants of rs12409277 had a significantly greater FFM% compared to TT homozygotes (*p* = 0.003).	Urano, et al., 2014. [70]

KE: knee extensor, HE: hip extensor, KF: knee flexor, HG: handgrip, FFM: fat-free mass, AFFM: appendicular fat-free mass, SMI: skeletal muscle index, CSA: cross sectional area, MVC: maximal voluntary contraction, TUG: timed up and go.

**Table 3:** Longitudinal studies on genetic association with muscle phenotypes.

Gene	Polymorphism	Study Design	Population Data	N	Muscle Phenotype	Results	Reference
*Hormone Genes*							
*RAMP3*	rs3757575 rs2074654 rs1294935 rs11982639 rs12702121	5- and 10-year follow-up	Swedish females (OPRA cohort) Aged 75 years Mean age 75.2 ± 0.1 years	1044	Body composition (total, legs and trunk FFM)	At baseline, C allele carriers of rs2074654 had significantly greater amounts of total and leg FFM (*p* = 0.041, 0.038 respectively) when compared to TT homozygotes. There were no significant associations at follow up.	Prakash, et al., 2019. [66]
*Growth Factor and Cytokine Genes*							
*IGF1*	192 bp allele	10-week intervention of single leg KE RT	Caucasians 32 males and 35 females Mean age 70 ± 6 years (males) and 67 ± 8 years (females)	67	Muscle strength (KE 1RM) Muscle volume (using CT) Muscle quality (1RM/muscle volume)	Carriers of the 192 allele achieved significantly greater KE 1RM improvements than non-carriers (*p* = 0.02). Although not significant, a trend towards greater muscle volume was noted between 192 carriers and non-carriers (*p* = 0.08).	Kostek, et al., 2005. [50]
*IGF1*	192 bp allele	10-week intervention of single leg KE RT	Blacks (12 males and 21 females) Whites (46 males and 49 females) Aged 50–85 years	128	Muscle strength (KE 1RM) Muscle volume (using CT) Muscle quality (1RM/muscle volume)	Significantly greater KE 1RM improvements were observed in individuals with ≥ 1 192 allele compared to non-carriers (*p* < 0.01). No significant differences in muscle volume or quality were noted.	Hand, et al., 2007. [43]
*Growth Factor and Cytokine Genes*							
*TNFα* *IL6* *IL10*	rs1800629 rs1800795 rs1800896	10-week intervention of either RT or AE	Brazilian females Aged ≥ 65 years 229 RT group and 222 AE group	451	Physical function (TUG and 10 m walking speed test)	Individuals homozygous for the G allele of polymorphism rs1800629 of TNFα achieved significantly greater TUG improvements with exercise compared to AA/AG genotypes (*p* < 0.001). A significant interaction was displayed between the 3 polymorphisms and TUG performance post exercise (*p* < 0.001). No significant interaction was observed between polymorphisms and 10 m walking speed test.	Pereira, et al., 2013. [65]
*Structural and Metabolic Genes*							
*ACE*	rs1799752 (I/D)	10-week intervention of unilateral KE RT	North Americans Whites (65%) and Blacks (35%) 86 males and 139 females Aged 50–85 years (mean age 62 years)	225	Body composition (FFM) Muscle volume (quadriceps) Muscle strength (KE 1RM)	At baseline, carriers of the DD genotype had significantly greater FFM than II homozygotes (*p* < 0.05). DD homozygotes also had greater baseline muscle volume in both the trained and untrained leg than II carriers (*p* = 0.02, 0.01 respectively). No significant associations were observed between genotypes and either 1RM or muscle volume adaptations to RT in either males or females.	Charbonneau, et al., 2008. [31]
*ACE*	rs1799752	12-month intervention of either PA or health education	Caucasians 97 males and 186 females Aged 70–89 years Mean age 77.2 ± 4.3 years	283	Physical function (400 m gait speed test and SPPB)	A significant difference was observed in gait speed and SPPB post PA in carriers of ≥ 1 D allele (*p* = 0.018, 0.015 respectively), but not in II homozygotes (*p* = 0.930, 0.275 respectively).	Buford, et al., 2014. [29]
*Structural and Metabolic Genes*							
*ACTN3*	rs1815739 (R577X)	10-week intervention of unilateral KE RT	Caucasians 71 males and 86 females Aged 50–85 years Mean age 65 ± 8 years (males) and 64 ± 9 years (females)	157	Body composition (FFM) Muscle volume (quadriceps) Muscle strength (KE 1RM, peak power and velocity)	At baseline, female XX homozygotes had significantly higher absolute and relative KE peak power and peak velocity than carriers of ≥ 1 R allele (*p* < 0.05). In males, change in absolute KE peak power post RT approached significance in RR homozygotes compared to XX carriers (*p* = 0.07). In females, change in relative KE peak power post RT was significantly higher in RR homozygotes compared to XX carriers (*p* = 0.02).	Delmonico, et al., 2007. [37]
*ACTN3*	rs1815739	5-year follow-up	White North Americans 726 males and 641 females (Health ABC cohort) Aged 70–79 years Loss to follow-up (372)	1367	Muscle volume (thigh muscle CSA) Muscle strength (KE isokinetic torque) Physical function (400 m walk test, SPPB, self-reported functional limitation)	At follow-up, male XX homozygotes had a significantly greater increase in 400 m walk time when compared to RX/RR carriers (*p* = 0.03). Female XX carriers had a 35% greater risk of functional limitation compared to RR homozygotes. No significant associations were noted between genotype and phenotypes at baseline in either males or females (*p* > 0.05).	Delmonico, et al., 2008. [38]
*Structural and Metabolic Genes*							
*ACE*	rs1799752 (I/D)	18-month intervention of exercise training (AE and RT)	Caucasians (75%), African-American (22%), Native American, Asian/Pacific Islander, Hispanic (3%)63 males and 150 femalesAged ≥ 65 yearsLoss to follow-up (37)	213	Muscle strength (concentric KE isokinetic strength) Physical function (6 min walk test, self-reported FAST)	Carriers of the DD genotype showed significantly greater improvements in concentric KE strength in response to exercise training than II homozygotes (*p* < 0.05). At baseline, no significant associations were noted between genotypes and measures of muscle strength and physical performance.	Giaccaglia, et al., 2008. [40]
*ACTN3*	rs1815739 (R577X)	Follow-up (NOSOS 1 year follow up, APOSS 2 year follow up)	Caucasian females (Scottish) NOSOS cohort (*n* = 1245) APOSS cohort (*n* = 2918) Mean age (NOSOS 69.6 ± 5.5 years and APOSS 54.8 ± 2.2 years)	4163	Fall incidences (self-reported for previous year)	In both NOSO and APOSS cohorts, baseline falls were significantly associated with carrying RX/XX genotypes (*p* = 0.049, 0.02 respectively). In a pooled analysis, follow-up fall incidences in the previous year were associated with X allele carriers (*p* = 0.01).	Judson, et al., 2011. [46]
*Structural and Metabolic Genes*							
*ACE*	rs1799752 (I/D)	Follow-up (4.1 year average)	Whites and Blacks 1439 males and 1527 females Aged 70–79 years	2966	Muscle volume (thigh muscle CSA) Muscle strength (maximal and mean isokinetic KE strength) Physical function (physical activity questionnaire, self-reported mobility limitations)	Among individuals with high levels of physical activity II homozygotes developed limitation at a 45% faster rate when compared to ID/DD carriers (*p* = 0.01). ACE genotype did not affect mobility limitation in inactive individuals, nor did it affect any other phenotype in either active or inactive individuals.	Kritchevsky, et al., 2005. [52]
*ACTN3* *ACE*	rs1815739 (R577X) rs1799752	24-week intervention of RT	Brazilian females Mean age 66.7 ± 5.5 years	246	Body composition (FFM, relative total FFM, AFFM and SMI) Muscle strength (KE isokinetic peak torque at 60°s)	At baseline, ACE DD homozygotes had significantly greater SMI than I/ID carriers (*p* = 0.044). ACTN3 X allele carriers had significantly more relative total FFM at baseline than RR homozygotes (*p* = 0.04). In response to RT, only ACE II homozygotes significantly increased AFFM (*p* < 0.001).	Lima, et al., 2011. [54]
*Structural and Metabolic Genes*							
*ACTN3* *ACE*	rs1815739 (R577X) rs1799752 (I/D)	12-week intervention of high-speed power training	Caucasian females Mean age 65.5 ± 8.2 years	139	Muscle strength (1RM bench press and leg extension and vertical jump) Physical function (sit-to-stand test)	Post intervention, ACE DD homozygotes showed significantly greater improvements in 1RM bench press and sit-to-stand tests (*p* = 0.019, 0.013 respectively) than II carriers. The same interaction approached significance for vertical jump (*p* = 0.052). ACTN3 RR homozygotes displayed significantly greater improvements across all measures than XX carriers (*p* < 0.05). At baseline, there were no significant differences between ACE or ACTN3 genotype for any phenotype.	Pereira, et al., 2013. [63]
*ACTN3* *ACE*	rs1815739 rs1799752	12-week intervention of high-speed power training	Caucasian females Mean age 65.5 ± 8.2 years	139	Muscle function (10 m maximal effort sprints, TUG test)	ACE DD homozygotes displayed significantly greater improvements in 10 m sprint time (*p* = 0.012) than II carriers, but not in GUG performance (*p* = 0.331). Similarly, ACTN3 RR homozygotes improved significantly more than XX carriers in 10m sprint time (*p* = 0.044) but not in TUG performance (*p* = 0.477). At baseline, there were no significant differences between ACE or ACTN3 genotype for any phenotype.	Pereira, et al., 2013 [64]
*Structural and Metabolic Genes*							
*ACEUCP2*	rs1799752 (I/D) rs659366	12-week intervention of RT, balance and cardiovascular exercises	Caucasians 18 males and 40 females Aged > 60 years Mean age 70.0 ± 5.9 years (males) and 69.7 ± 5.3 years (females)	58	Muscle strength (HG strength) Physical function (30 s sit to stand, 30 s bicep curls, 8 ft TUG, 6 min walk, Purdue pegboard test)	At baseline, ACE II homozygotes performed significantly worse than ID/DD carriers in the 6 min walk and 8 ft TUG tests (*p* = 0.008, *p* < 0.001 respectively). GG carriers of rs659366 performed significantly worse in the 8 ft TUG test compared with AA/GA genotypes (*p* = 0.045). Post intervention, GG carriers of rs659366 had the greatest improvements in 8 ft TUG performance compared to AA/GA carriers (*p* = 0.023), while a trend for greater improvements in bicep strength was noted for ID/DD carriers compared to II carriers (*p* = 0.099).	Keogh, et al., 2015. [47]
*APOE*	rs7412 rs429358 (e4 status)	6-year follow-up	Caucasians (Dutch) 553 males and 709 females Aged > 65 years Mean age 74.9 ± 5.8 years Loss to follow-up (449)	1262	Physical function (5 chair stand test, 3 m gait speed, self-reported mobility)	At baseline, e4 carriers displayed significantly worse gait speed and chair stand performance (*p* = 0.006, 0.015 respectively) than the e3 group. At follow-up, e4 status was associated with significantly worse chair stand performance (*p* = 0.034) compared to e3 carriers.	Melzer, et al., 2005. [60]
*Structural and Metabolic Genes*							
*APOE*	rs7412 rs429358 (e4 status)	4-year follow-up	Swedish 245 males and 364 females Aged 75 years Loss to follow-up (28)	609	Muscle strength (HG strength) Physical function (20 m maximum gait speed, 5 chair stand test, 30 s single leg stand)	Subjects who carried the APOE e4 allele had a significantly larger decline in HG between age 75 and 79 compared to non-carriers (*p* = 0.015). Carriers of the APOE e4 allele had significantly lower HG at age 79 compared to non-carriers (*p* = 0.006). The effect of e4 allele on HG was significantly larger at age 79 than age 75 (*p* = 0.033).	Skoog, et al., 2016. [68]
*APOE*	rs7412 rs429358 (e4 status)	Follow-up (3-year average)	North Americans (67.8% White and Blacks 27.1%) 235 males and 392 females Mean age 79.4 ± 5.2 years	627	Physical function (15 ft and 20 ft gait speed, disability scale examining ability to perform ADL’s)	Males carrying the ε4 allele showed a significantly more rapid decline in gait speed than male non-carriers (*p* = 0.04). This was most significant in white males only (*p* = 0.007). Similarly, males who carried the e4 allele had a significantly greater risk of disability than non-carriers (*p* = 0.007).	Verghese, et al., 2013. [71]
*Structural and Metabolic Genes*							
*ZNF295* *C2CD2*	rs928874 rs1788355	GWAS 2-year follow-up	Italians ilSIRENTE cohort (*n* = 286) 116 males and 170 females Mean age 86.1 ± 4.9 years Replication cohort inCHIANTI (*n* = 1055) 440 males and 615 females Mean age 67.8 ± 15.7 years	1341	Body composition (calf circumference, mid-arm muscle circumference) Muscle strength (HG strength) Physical function (4 m walk test, SPPB, ADL)	In the ilSIRENTE cohort, rs928874 and rs1788355 were significantly associated with 4 m gait speed (*p* = 5.61 × 10^−8^, 5.73 × 10^−8^ respectively). This association was not replicated in the inCHIANTI cohort.	Heckerman, et al., 2017. [44]

KE: knee extensor, HG: handgrip, FFM: fat-free mass, AFFM: appendicular fat-free mass, SMI: skeletal muscle index, RT: resistance training, AE: aerobic exercise, CT: computed tomography, CSA: cross sectional area, 1RM: 1 repetition maximum, PA: physical activity, TUG: timed up and go, ADL: activity of daily living, SPPB: short physical performance battery.
